# Amphiphilic Block Copolymer Poly (Acrylic Acid)-B-Polycaprolactone as a Novel pH-sensitive Nanocarrier for Anti-Cancer Drugs Delivery: In-vitro and In-vivo Evaluation

**DOI:** 10.3390/polym11050820

**Published:** 2019-05-07

**Authors:** Huanhuan Liu, Hong Chen, Fuhu Cao, Daiyin Peng, Weidong Chen, Chuanling Zhang

**Affiliations:** 1 The College of Pharmacy, Anhui University of Chinese Medicine, Hefei 230012, China; huanhuanliu2016@ahtcm.edu.cn (H.L.); chong19960320@126.com (H.C.); pengdy@ahtcm.edu.cn (D.P.); 2 Anhui Province Key Laboratory of Advanced Catalytic Materials and Reaction Engineering, School of Chemistry and Chemical Engineering, Hefei University of Technology, Hefei 230009, China; fhcao@hfut.edu.cn

**Keywords:** amphiphilic block copolymer, gambogenic acid, pH-sensitive

## Abstract

Gambogenic acid (GNA) has been demonstrated with outstanding antitumor activity as a potential antitumor drug in recent years. However, the low solubility and deficient bioavailability of GNA seriously hinder its practical application in the clinic area. In this study, a novel amphiphilic block copolymer, poly (acrylic acid)-*b*-polycaprolactone (PAA-*b*-PCL) is prepared and assembled into pH-responsive polymeric micelles (PMs) as one mold of drug delivery system (DDS) with unique properties. Relevant investigation on PMs exhibits excellent carrying potential and pH-dependent release performance for GNA. The drug loading capacity (DLC) and drug loading efficiency (DLE) for GNA-loaded PMs can be achieved as high as 15.20 ± 0.07% and 83.67 ± 0.49%, respectively. The in vitro experiments indicate that the GNA releasing time, cytotoxicity, and cellular uptake are significantly enhanced. Especially, the peak concentration (Cmax) and area under the curve (AUC) are promoted sharply in the GNA-loaded PMs concentration-time curve. This study not only provides a novel way to widen the application of anticancer GNA in the future, but also extends the potential of stimuli-responsive copolymers to biomedical applications.

## 1. Introduction

Gambogenic acid (GNA), as a kind of weakly acidic ingredient, extracted from Chinese traditional medicine Gamboge, has been proved with outstanding inhibition on proliferation and growth of various tumor cell [1,2,3]. Compared with other basic anticancer drugs (doxorubicin, paclitaxel, Camptothecin, et al.) in the clinic, GNA can be much easier to cross the tumor cell membrane in the form of molecules [4]. However, great limits have been found in its potential applications, such as inflammation to blood vessel, low solubility, deficient bioavailability, or property of toxicity [5,6]. To conquer these problems mentioned above, much effort has been devoted to explore effective carriers, such as PEGylated Niosomes [7], solid lipid nanoparticles [8], and nanostructured lipid carriers [9]. Nevertheless, the drug loading efficiency (DLE) and drug loading capacity (DLC) of nanocarriers obtained through those strategies are still limited.

Polymer micelles (PMs), composed of an outer hydrophilic shell and inner lipophilic core, have appealed arousing attention due to its unique performances when performed as nanocarriers, for example, improved drug encapsulation ability and lipophilic drug solubility exceeding other molds of nanocarriers [10,11,12,13]. Jiang et al. reported that supramolecular copolymer micelles composed of poly(DL-lactide) as hydrophobic core and 7-armed star poly(2-methyl-2-oxazoline) as hydrophilic shell could be used as carriers of cabazitaxel with excellent drug loading content (17.5%) [14]. However, the therapeutic efficacy of traditional micelles was queried because of the laggard biodegradation of the nanoparticles, extending the time between the encapsulated drugs being released from micelles and arriving at the tumor site. Recently, stimuli-responsive PMs have exhibited great promise on the enhancing efficiency of drug delivery [15,16]. For instance, Chen et al. developed polymer micelles by PEG and poly (L-lactide) with remarkable pH-dependent association/dissociation, which had been successfully employed as carriers for doxorubicin (DOX) with significantly improved antitumor efficacy [17]. Ji et al. constructed polymeric prodrug micelles with acid and near-infrared (NIR) light dual-responsive properties through the strategy of introducing photo-sensitizer IR-780 in the pH-responsive polymer micelles for the effective drug delivery system, showing fast DOX release in acid condition and effective hyperthermia effect when exposed to NIR laser [18]. Wang et al. reported intelligent PMs system of Mpeg-P (TPE-co-AEMA) copolymer with pH and redox dual-responsive properties for bioimaging and smart drug release behaviors, such as high loading and enhanced efficacy of drugs [19]. Among the various stimuli, the pH-stimulus is relative popular due to its broad application in anticancer drugs delivery [20,21,22,23,24,25]. There are numerous differences between tumor tissues and the normal, such as pH varying in a wide range from about 7.4 (physiological), 6.8 (extracellular in tumor tissues), 5.5–6.0 (endosomes), to 4.5–5.0 (lysosomes). Therefore, exploration of novel pH-responsive PMs with controlled drug release behavior for GNA has a very important significance and would meanwhile exhibit great promises for effective cancer therapy.

In recent years, bio-adhesive poly (acrylic acid) (PAA) and poly (methacrylic acid) (PMAA) have been demonstrated to be the representative pH-sensitive segments performed in stimuli-responsive systems [26]. For example, Wang et al. reported a smart nanocarrier based on a poly (acrylic acid-co-spiropyran methacrylate) nanogel for delivery anticancer drugs, which could provide chemotherapy and fluorescence imaging to cancer cells [27]. Liu et al. developed poly (methacrylic acid)/poly (N-isopropylacrylamide) (PMAA/PNIPAM) to form independent pH and temperature dual-stimuli responsive microgels, which showed excellent drug-loading efficiency and smart controlled release performance [28]. As a member of polyesters, polycaprolactone (PCL) possessing distinctive advantages of biodegradable and miscible properties had been found to be significant in the medicinal applications and would be an outstanding candidate as the hydrophobic block in the amphiphilic polymer [29]. Hence, the functional amphiphilic block copolymers with PAA and PCL segments used for medical application is highly desired.

**Scheme 1:**
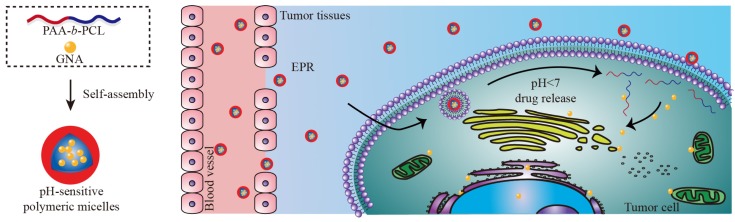
Schematic illustration of the fabrication of pH-sensitive Polymer Micelles and GNA release from the micelles for cancer therapy.

In this study, we have developed a novel amphiphilic block copolymer poly (acrylic acid)-*b*-polycaprolactone (PAA-*b*-PCL) synthesized via successive reactions of reversible addition-fragmentation chain transfer (RAFT), ring-opening polymerization (ROP) and hydrolytic reaction. The target copolymer could be self-assembled into a pH-sensitive nanoparticle for the delivery of anticancer drug GNA (Scheme 1). On this basis, drug-loading, in vitro release, cytotoxicity, intracellular uptake, and pharmacokinetic properties from GNA-loaded PAA-*b*-PCL micelles were investigated systematically for evaluation. Noteworthy is that this study was the first report on the GNA delivery system based on pH-responsive copolymers, revealing great potential in smart Chinese Medicine delivery systems.

## 2. Materials and Methods

### 2.1. Materials

The generation of GNA was obtained by extraction and isolation from the gamboge’s resin (98%). ε-Caprolactone (CL, 99%) was treated with distillation under reduced vacuum. Tert-butyl acrylate (*t*BA, 98%, Alfa Aesar) was purified through a basic alumina column. Stannous octoate (Sn (Oct) 2, 97%) was used as received. 2,2′-azobis (isobutyronitrile) (AIBN) was recrystallized twice from ethanol. Prop-2-ynyl-3-(5-cyano-5-phenylthiocarbonylsulfanyl) pentanoyloxy-2-(2,2-dihyd -roxymethyl)-propionyloxymethyl-2-methylpropanoate (PCBP) was prepared and purified in reference to literature procedures [30].

### 2.2. Preparation of PAA-b-PCL

First, *t*BA (3.67 g, 28.6 mmol), PBCP (0.200 g, 0.286 mmol) and AIBN (0.009 g, 0.057 mmol) were dissolved in toluene (9.5 mL). The contents were flushed with nitrogen for 20 min followed by polymerization at 60 °C for 20 h. The solution was cooled down and precipitated into the mixed solution of methanol and deionized water for three times. 2.55 g of P*t*BA (64.1% conversion) was obtained after vacuum drying. Gel permeation chromatography (GPC) was carried out to estimate apparent molecular weight (M_n,GPC_) and polydispersity (PDI), ^1^H NMR was performed to determine number-average molecular weight (M_n,NMR_), the results of GPC and ^1^H NMR analysis were M_n,GPC_ = 9880 g mol^−1^ and PDI = 1.12. M_n,NMR_ = 11100 g mol^−1^. And P*t*BA:^1^H NMR (CDCl_3_): δ7.92, 7.57, 7.40 (m, Ph*H*), 4.74 (s, 2H, C*H*_2_O), 2.3–2.8 (m, 5H, CH and C*H*_2_CH2COO), 2.23 (m, CH_2_C*H* of P*t*BA), 0.6–2.0 (m, C*H*, C*H*_2_ and C*H*_3_ resulting from CTA, C*H*_3_ of P*t*BA). Second, the prepared P*t*BA (1.406 g, 0.200 mmol), CL (1.136 g, 10.0 mmol) and Sn (Oct)_2_ (0.041 g, 0.100 mmol) were also dissolved in toluene and total volume was 3.3 mL. With little change, the procedures were performed under nitrogen. The reactor was degassed by three freeze-pump-thaw cycles to remove the residual gas within reaction solution and then heated to 110 °C for polymerization. After 20 h, the prepared polymer solution was precipitation into hexane and 1.86 g of P*t*BA-*b*-PCL diblock copolymer with 39.6% conversion was obtained. In this part, GPC and ^1^H NMR analyses: M_n,GPC_ = 13,700 g mol^−1^, PDI = 1.20, M_n,NMR_ = 26,100 g mol^−1^. P*t*BA-*b*-PCL: ^1^H NMR (CDCl_3_): δ 7.92, 7.57, 7.40 (m, Ph*H*), 4.74 (s, 2H, C*H*_2_O), 4.06 (t, C*H*_2_O of PCL), 2.31(t, C*H*_2_CO of PCL), 2.23 (m, CH_2_C*H* of P*t*BA), 0.6–2.0 (m, C*H*, C*H*_2_ and C*H*_3_ resulting from CTA, C*H*_2_ of PCL and C*H*_2_, C*H*_3_ of P*t*BA). Last, the hydrolysis of P*t*BA-*b*-PCL copolymer (0.50 g) was conducted in 15 mL of dichloromethane and trifluoroacetic acid (0.25 mL) were added. The reaction was maintained in room temperature for 40 h. After concentration, precipitation, and drying, 0.40 g PAA-*b*-PCL diblock copolymer was isolated.

### 2.3. Formation of Blank and GNA-Loaded PMs

1.0 mL of dimethyl-sulphoxide (DMSO) containing 21.0 mg PAA-*b*-PCL was added dropwise into of distilled water (14.0 mL) followed with vigorous stirring. 3 h later, the mixture was transferred into a dialysis bag (MWCO 3500) to remove the residual DMSO and complete blank PMs could be obtained after 24 h. The diameter of blank PMs was analyzed by dynamic light scattering (DLS, Zetasizer Nano-ZS, Malvern Panalytical, Malvern, England) and transmission electron microscope (TEM, Hitachi H-600, Hitachi, Japan) was applied to investigate the morphology. The preparation for GNA-loaded PMs was similar with that of blank PMs, but the only difference was that DMSO (1.0 mL) was needed. The concentration of GNA was measured by UV-vis spectrophotometry (UV-2550, Shimadzu, Japan) with the measuring wavelength at 360 nm. On the basis of UV-vis analysis, the DLC and DLE of micelles could be determined through the following equations:

DLC (wt.%) = m_loading drug_/m_total amoumt of micelles_ × 100% (1)

DLE (wt.%) = m_loading drug_/m_drug in feed_ × 100% (2)

### 2.4. pH-Triggered Reassembly of PMs and GNA-Loaded PMs Releasing Behavior

The reassembled behavior of PMs in response to pH was monitored by DLS and TEM, respectively. A final solution with pH 5.3 was achieved by direct dripping of 0.1 M HCl into 2.0 mL of blank and GNA-loaded PMs solution, followed by the stirring the solution at 37 °C. GNA-loaded PMs were subjected to show the GNA releasing behavior in vitro. Typically, free-GNA and GNA-loaded PMs solution (5 mL) were separately filled into a dialysis bag (MWCO 8000–14,000) and placed into 40 mL of prepared media consisted of phosphate buffered saline (PBS, pH = 7.2) and 1% (v/v) Tween 80 at 37 °C. 200 μL of materials were sampled and same volume of fresh medias were refilled in various time points (2, 4, 6, 8, 12, and 24 h). Then, the samples were achieved through centrifugation at speed of 12,000 rpm/min and measured by high performance liquid chromatography (HPLC). Finally, the cumulative release profiles could be plotted.

### 2.5. Cell Viability Assay (MTT)

HepG2, with a density of 3 × 10^4^ cells for each well, was seeded at 96-well plates and cultivated in 100 μL of fresh DMEM medium. The cell viability was detected by the MTT assay. An atmosphere with 37 °C and 5% CO_2_ were provided to keep cells growth. 24 h later, the cells containing varied concentrations of free-GNA, blank and GNA-loaded PMs were exposed to 100 μL of fresh mediawhich for 24 h. Then, each well was added with 20 μL of 3-(4, 5-dimethylthiazol-yl)-2 and 5-diphenyltetrazolium bromide (MTT) solution (5 mg/mL) and stained for 4 h. 150 μL of DMSO was employed to remove the medium and dissolved the formazan. At last, the cell viability determined by the optical density (OD) was measured at 490 nm by a multilabel counter.

### 2.6. Cellular Uptake

6-well cell culture plates were seeded with HepG2 cells (3 × 10^5^ cells/well) and then incubated in 1 mL of medium at 37 °C under atmospheres of 5% CO_2,_ respectively. After being incubated for 48 h, the medium was removed and an equal volume of flesh medium (containing 10 μg free-GNA or GNA-loaded PMs) was added to co-culture with HepG2 cells for 5, 15, 30, 45, 60, 120, and 240 min, respectively. At the predetermined time point, the drug-contained medium was removed and washed with 4 °C PBS directly for three times. Subsequently, the samples were lysed by cell lysis solution and then the cell lysate granted was stored under refrigeration at −20 °C. The intra-cellular GNA contents were determined by HPLC.

### 2.7. In Vivo Studies of GNA-Loaded PMs

Two randomized groups of Sprague-dawley (SD) rats were designed, of which six healthy male SD rats (Laboratory Animal Center of Anhui Medical University, Hefei, China) weighing from 200 to 220 g were included. GNA and GNA-loaded PMs solution with certain concentration (equivalent to a 1.5 mg/Kg GNA dose) were administrated to rats via the lateral tail vein. Then the blood was collected from orbital plexus at prespecified time points (3, 5, 10, 30, 45, 60, 120, 240 min). The pharmacokinetic parameters of GNA were determined by DAS 2.0. This animal experiment has followed the National Institutes of Health guide for the care and use of Laboratory animals.

### 2.8. Statistical Analysis

All results mentioned in this study were represented as mean ± S.D. Additionally, SPSS 17.0 was carried out for statistical analyses and *t*-test or ANOVA analysis were included. When the value of *p was lower than 0.05, the level of significance could be accepted.

### 2.9. Characterization

The TEM images were carried out on a Hitachi H-600 transmission electron microscope. The M_n,GPC_ were investigated on a Waters 150-C GPC (bead size of 10 μm in the ultrastyragel columns, THF as eluent at a flow rate of 1.0 mL min^−1^) at 35 °C. The samples were calibrated with the standard samples. The ^1^H NMR analyses were conducted on a Varian spectrometer (400 MHz, Varian, Walnut Creek, CA, USA) at 25 °C using CDCl_3_ as a solvent. The DLS analyses were performed on a Zetasizer Nano-ZS (Malvern Instruments, Malvern, England) equipped with He-Ne laser (633 nm) using back-scattering detection. The FT-IR spectra were measured on a Perkin-Elmer 2000 spectrometer using KBr discs.

## 3. Results and Discussion

### 3.1. Synthesis and Characterization of PAA-b-PCL

**Figure 1:**
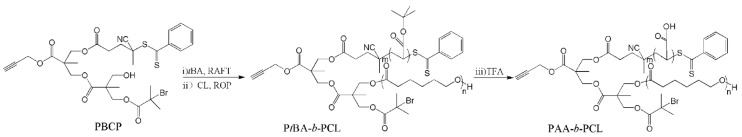
Synthetic Scheme of pH responsive diblock copolymer PAA-*b*-PCL.

**Figure 2:**
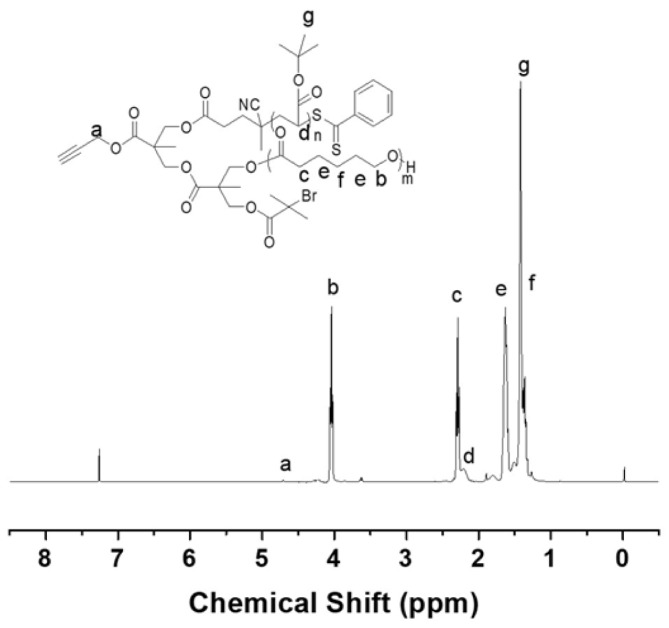
^1^H NMR spectrum of P*t*BA-*b*-PCL in CDCl_3_ (δ_solvent_ = 7.26 ppm).

A multifunctional agent PCBP with alkyne, bromide, dithiobenzoate and hydroxyl groups was employed to synthesize P*t*BA-b-PCL diblock copolymer. Alkyne and bromide functionalized PtBA-*b*-PCL diblock copolymer could be achieved by successive RAFT and ROP polymerization (Figure 1). As shown in Table 1, P*t*BA with well-controlled molecular weight and relatively low PDI was obtained with PCBP as a functional initiator in the RAFT polymerization. In the ^1^H NMR spectrum of P*t*BA, 7.92, 7.57, 7.40 (Ph*H*) were the characteristic resonance signals of PCBP initiator and 2.23 (CH_2_C*H* of P*t*BA) and 1.44 ppm (C*H*_3_ of P*t*BA) were the resonance signals of P*t*BA segment (Figure S1). By comparing integration areas at 2.23 (CH_2_C*H*) and 4.74 ppm (C*H*_2_O), the M_n,NMR_ was determined to be 11,100 g mol^−1^ and M_n,GPC_ and PDI were estimated to be 9880 g mol^−1^ and 1.12 by GPC analysis. To obtain a P*t*BA-*b*-PCL diblock copolymer, ROP polymerization of CL was mediated by introducing P*t*BA (as a macro CTA). In ^1^H NMR spectra (Figure 2), 4.06 (C*H*_2_O) and 2.31 ppm (C*H*_2_CO) were the typical resonance signals of the PCL block and 2.23 ppm (CH_2_C*H*) were represent the P*t*BA block. Besides, the M_n,NMR_ values were determined by comparing integration areas of characteristic protons of C*H*_2_O (4.74 ppm) and C*H*_2_CO (2.31 ppm for PCL). The result were shown that there were only little difference between M_n,NMR_ and M_n,GPC_ values and the PDI indices were relatively low, with GPC traces wholly shifted to the higher molecular weight side (Figure S2), which reveals that the chain extension polymerization was performed with high efficiency. To obtain the targeted PAA-*b*-PCL diblock copolymer, a selective hydrolysis of the P*t*BA segment was performed in dichloromethane. Additionally, the IR spectrum of PAA-*b*-PCL as shown in Figure S3, the absorption bands of various segments appeared at 1783 (C = O of PAA) and 1729 cm^−1^(C = O of PCL). To sum up, the results of GPC, ^1^H NMR and FT-IR were employed to confirm that a PAA-*b*-PCL block copolymer with well-controlled chemical compositions was successfully synthesized and the selective hydrolysis could efficiently achieve.

### 3.2. Formation and Stimuli-Triggered Morphological Transition of PMs

**Figure 3:**
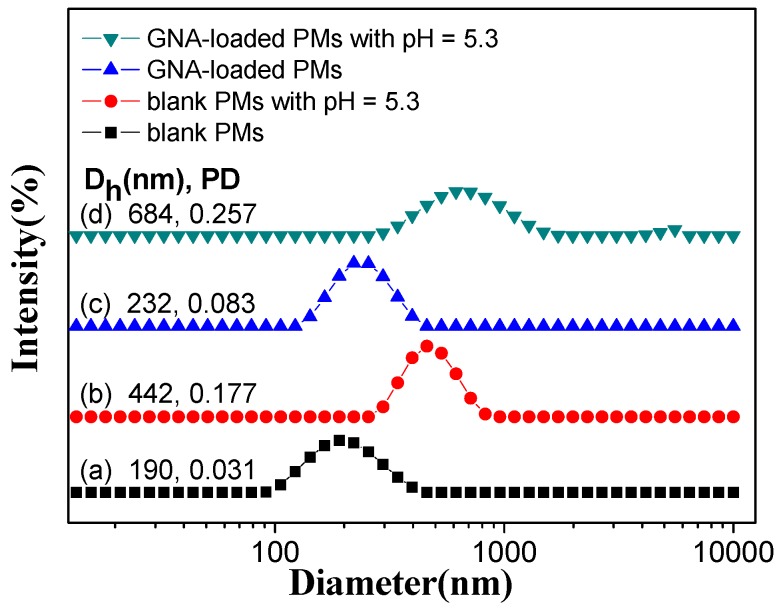
Influence of pH on DLS plots of PCL-*b*-PAA aggregates (c = 1.4 mg mL^−1^) (a and b) and treated with HCl (0.1 mol L^−1^) for 2 h (b and d).

**Figure 4:**
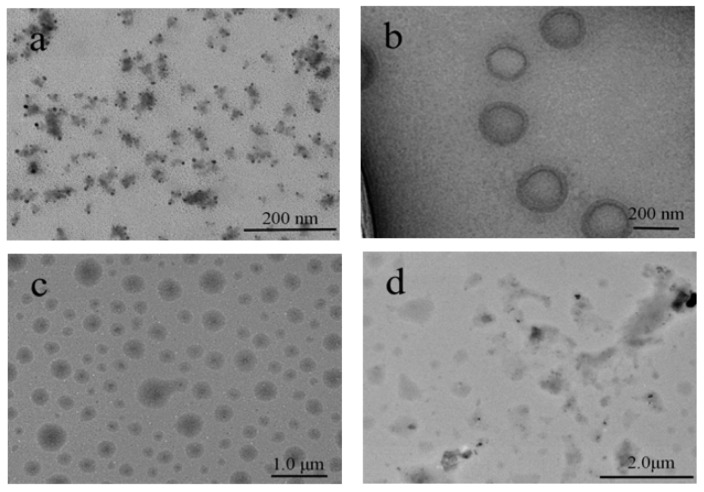
TEM images of PCL-*b*-PAA aggregates (c = 1.4 mg mL^−1^) in different environment: (**a**) Blank PMs at neutral environment; (**b**) blank PMs at weakly acid environment; (**c**) GNA-loaded PMs at neutral environment; and (**d**) GNA-loaded PMs at weakly acid environment.

To show the impact exerted by pH on polymer aggregate behaviors, amphiphilic block copolymer PCL-*b*-PAA was selected as a standard sample. The pH-dependent size variations of PCL-*b*-PAA aggregates at different buffer solution were investigated by DLS, in which hydrodynamic diameter (D_h_), the highest intensity of peak size (D_peak_), distribution of particle size (PD) could be easily observed. Merely the unimodal distribution of the micelles was indicated in DLS plots (Figure 3) and it was attained that the particle parameters (D_h_, D_peak_, PD) of these aggregates were 190 nm, 208 nm, and 0.031 (blank PMs in pH 7.2), 232 nm, 244 nm and 0.083 (GNA-loaded PMs in pH 7.2), 442 nm, 484 nm, and 0.177 (blank PMs in pH 5.3) and 684 nm, 706 nm and 0.257 (GNA-loaded PMs in pH 5.3). Corresponding TEM images revealed that PCL-*b*-PAA aggregates (pH 7.2, Figure 4a) and GNA-loaded PMs (pH 7.2, Figure 4c) were multicompartment micelles and spherical micelles, respectively. And the results were showed that the hydrodynamic diameters of blank PMs estimated by DLS and TEM were completely different (D_h,TEM_ ≈ 90nm and D_h,DLS_ = 190 nm). As is well known, a dried nanostructure would be shrunk significantly. The presence of hydrogen-bonding interactions among PCL and PAA and the bulky building blocks tended to form co-aggregates, while the filling of polymer chains was looser [31].

Based on the pH-sensitive behavior of PAA-*b*-PCL, pH-triggered morphological transition of blank or GNA-loaded aggregates were preliminarily investigated. With buffer solution changing from neutral to weakly acidic, the morphology of PAA-*b*-PCL evolved from multicompartment micelles to spherical vesicle (Figure 4a,b), which explained by the synergistic effect of increased hydrophobicity of PAA chains at a lower pH and various strength and proportion of inter-and intra-molecular interactions, such as hydrophobic and hydrogen-bonding interactions. With the pH of buffer solution being decreased to 5.3, significant increases on size and size distribution were found for the GNA-loaded aggregates (Figure 3c,d). Further destruction of the micelle core forces the micelle to be dissociated and aggregated into a larger formation (Figure 4d) [32].

### 3.3. DLC, DLE and In Vitro Releasing Behavior

**Figure 5:**
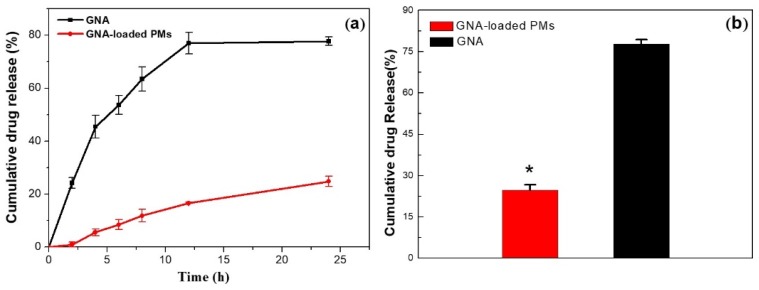
In vitro release studies of GNA from PMs. (**a**) GNA cumulative release in PBS at pH 7.4; (**b**) total release of different GNA formulation after 24 h. * *p* < 0.05. (Mean ± S.D., *n* = 3).

The DLE of GNA-loaded PMs was 83.67 ± 0.49% and the DLC was achieved to 15.20 ± 0.07%, which was the highest among the nanocarrier for GNA reported before [7,8,9]. Then the release kinetics of GNA encapsulated by the polymeric particles was measured by a dialysis method (37 °C, pH 7.2). As shown in Figure 5a, free-GNA were released about 77.02% within 12 h and then a plateau was reached. Conversely, the GNA released from micelle systems was found to be much slower, only16.64% of GNA were released within the first 12 h, which could be concluded that the speed of GNA releasing in PMs was significantly lower than that of free-GNA (Figure 5b). These results might originate from the hydrophobic GNA being preferred to retain in the micelles. Hence, it could be inferred that the GNA-loaded PMs could keep a prominent stability in the course of systematic circulation.

### 3.4. Cytotoxicity and In Vitro Cellular Uptake Assays

**Figure 6:**
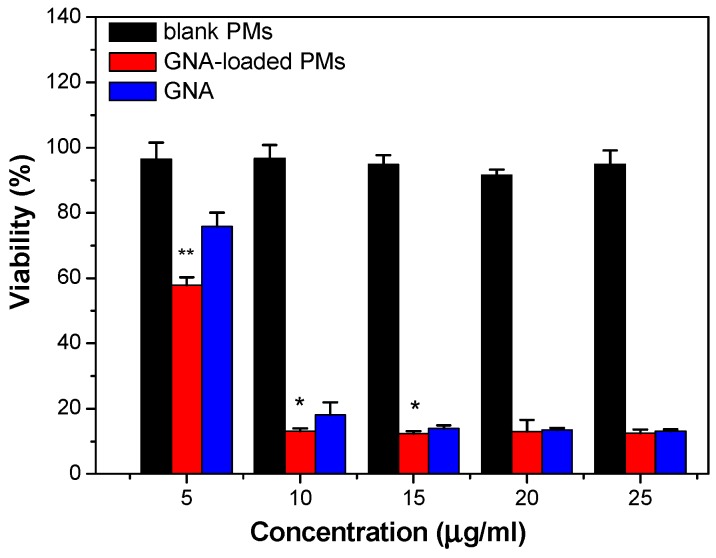
Cell viability of HepG2 cells incubated with GNA, GNA-loaded PMs and blank PMs at various concentrations at 37 °C for 24 h. Error bars denote standard deviations from six parallel trials * *p* < 0.05, ** *p* < 0.01.

Cytotoxicity of PAA-*b*-PCL and different GNA formulation was estimated through adopting MTT assay. Previous reports have proved that GNA could efficiently inhibit the growth of cell tumor, such as human hepatoma cell. In this study, HepG2 cells as the most common cells in the study of anti-cancer activity were employed and treated with GNA in the research. As shown in Figure 6, after cultured with the blank micelles in the concentrations ranging from 23.33 to 116.67 μg/mL, cell viabilities of HepG2 still remained at 95%, indicating that PCL-*b*-PAA was almost no-toxicity at this dose. Therefore, PCL-*b*-PAA could be a safe delivery tool with prominent biocompatibility. Not surprisingly, the cytotoxicity was improved with the amount of GNA increasing, both the free and loaded in PMs (Figure 6). And the half-maximal inhibitory concentration (IC50) values of GNA-loaded PMs and the free GNA could be seen in Table 2, the GNA-loaded PMs was lower, which might be due to the increased cellular uptake of GNA by HepG2 [33,34]. This speculation could be confirmed by the cell viability change. When the amount of GNA was more than 10 μg mL^-1^, there were no obvious cytotoxicity change in GNA-loaded PMs with increasing dose, but the free GNA could further decline until the final concentration reached 15 μg mL^-1^. Those results of cytotoxicity tests were indicated that the encapsulation of GNA into PCL-b-PAA micelles could increase the cytotoxicity of GNA and the same therapeutic effect would be achieved with the reduced dosage of GNA-loaded PMs when it comes to tumor treatment.

**Figure 7:**
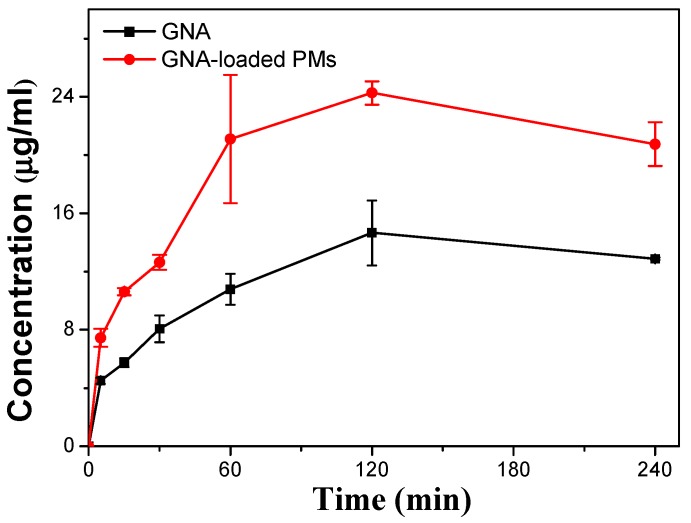
Effect of time on the cellular uptake of GNA in Hep G2 cells. Hep G2 cells were incubated with GNA and GNA-loaded PMs (10 μg of GNA) for 5, 15, 30, 45, 60, 120, and 240 min, respectively. Intracellular GNA was assayed by HPLC (Mean ± S.D., *n* = 3).

To evaluate GNA-loaded PMs and the free PMs further, in vitro cellular uptake assays were carried out and the efficiency of intracellular drug uptake was estimated through HPLC (Figure 7). The maximum amounts of GNA in cells incubated with GNA-loaded PMs and free-GNA were achieved at the same time (120 min) and the GNA-loaded PMs was much higher than that of free-GNA (24.27 versus 14.66 μg mL^-1^). It could be concluded that the intracellular uptake was increased when GNA was encapsulated in PMs and the GNA-loaded PMs with higher cytotoxicity could be also well interpreted. Previous literatures have proved that PMs have similar structures with the cell membranes, which would help PMs to pass through cell membranes and promote intracellular accumulation of drugs [35,36,37]. Besides, there were many other factors to promote the cellular uptake efficiency of PMs. For instance, micelles with size over 20 nm were internalized via multiple pinocytosis pathways [38]. As time went by, the concentration of GNA in the HepG2 were decreased after 2 h, which could be explained by the metabolized and eliminated GNA in the cell being higher than absorbed.

### 3.5. Pharmacokinetic Study

**Figure 8:**
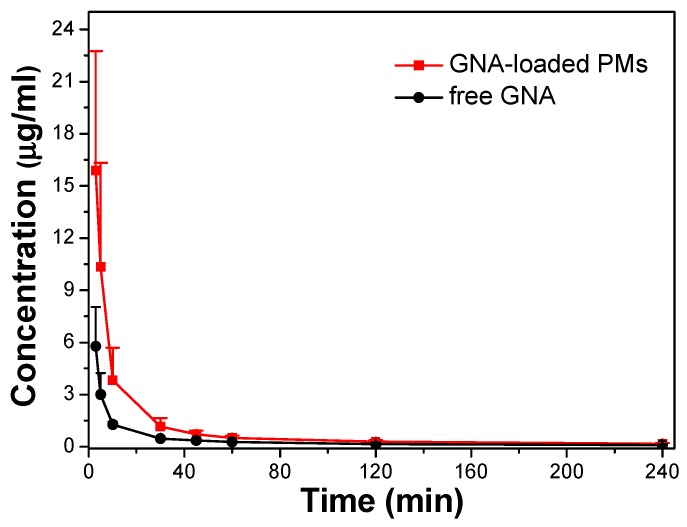
Mean plasma concentration-time profile of GNA in rats after intravenous administration GNA solution, GNA-loaded PMs at a single dose of 1.5 mg kg^−1^ (Mean ± S.D., *n* = 6).

The quality of GNA-loaded PMs was furtherly evaluated by pharmacokinetic study. Relative pharmacokinetic parameters were obtained through a non-compartment model and shown in Table 3, the GNA concentration in plasma were presented by mean ± S.D. and illustrated in Figure 8, respectively. As indicated for overall time points, the GNA plasma concentrations outstripped those performed with GNA solution in rats performed with GNA-loaded PMs (*p* < 0.05). Bioavailability, an indicator used to evaluate the clinical potential of drugs, could be associated with the area under the curve (AUC). The results were shown that AUC0→*t* values of GNA-loaded PMs were 3.94 times than that of free-GNA, therefore PMs could promote the bioavailability sharply. In addition, the peak concentration (Cmax) value of GNA-loaded PMs was 2.75 times higher than GNA, which could be attributed to that free-GNA without protection was fast metabolized after the drug injected into rats for 3 min (the first take blood point). Not surprisingly, the MRT of GNA-loaded PMs was declined slightly with no statistically marked, which could be inferred that micelles were absorbed into the blood after performance [39]. Briefly, the result of pharmacokinetic study proved that the PMs could contribute to the circulation of GNA in body. The results of this part could be clearly observed that the time GNA existed in the plasma was much shorter than that of drug releasing. The possible mechanisms for the difference between the vitro and vivo could be that: (1) The steady structure of micelles might be destroyed by the numerous ingredients in the plasma; (2) the blood vessels could be passed through micelles, but dialysis bags cannot; and (3) the area of blood vessels was much larger than that of the dialysis bags.

## 4. Conclusions

In summary, a novel amphiphilic block copolymer poly (acrylic acid)-*b*-polycaprolactone (PAA-*b*-PCL) with excellent pH-triggered morphological transition performance has been successfully synthesized through successive reactions of RAFT, ROP, and hydrolytic reaction. The target copolymer has been found with well-controllable chemical composition and a low polydispersity from the analyses of GPC, ^1^H NMR, and FT-IR. The segment of PAA as the hydrophilic shell in the micelle is the key to its pH-responsive performance and the segment of PCL as a biodegradable block plays a significant role on its biocompatibility. Furthermore, the nanoparticles self-assembled by the copolymer delivers the highest DLE and DLC value (83.67 ± 0.49% and 15.20 ± 0.07%) and the slow and stable releasing behavior (16.64% within the first 12 h) when performed as the nanocarrier for GNA. Our preliminary experiments indicated that the encapsulation by micelles could enhance both the cytotoxicity and the circulation of GNA in the body, providing great potential in smart Chinese Medicine delivery systems for tumor treatment further.

## Figures and Tables

**Table 1:** Results for synthesis of P*t*BA and P*t*BA-*b*-PCL diblock copolymer *^a^.*

Run	Polymer	I	M	Dp_0_	C *^b^*	M_n,th_ *^c^*	M_n,gpc_ *^d^*	Pdi *^d^*	M_n,nmr_ (dp_pm_) *^e^*
1	P*t*BA	PCBP	*t*BA	100	0.641	8920	9880	1.12	11,100 (81)
2	P*t*BA-*b*-PCL	P*t*BA	CL	50	0.480	10,720	13,700	1.20	26,100 (132)

*^a^* Reaction conditions: [*t*BA]_0_:[PCBP]_0_:[AIBN]_0_ = 100:1:0.2, [M]_0_ = 3.0 mol L^−1^ (run 1); [CL]_0_:[P*t*BA]_0_:[Sn (Oct)_2_]_0_ = 50:1:0.5, [M]_0_ = 1.0 mol L^−1^ (run 2). *^b^* Monomer conversion (C, runs 1–2) determined by gravimetry. *^c^* Theoretically calculated molecular weight. *^d^* Number-average molecular weight estimated by GPC (*M*_n,GPC_, runs 1–2). *^e^* Number-average molecular weight determined by ^1^H NMR analysis.

**Table 2:** IC_50_ (μg/mL) of HepG2 incubated with GNA solution and GNA-loaded PMs (*n* = 6).

Formulation	GNA Solution	GNA-Loaded PMs
IC_50_ (μg/mL)	7.029	5.040

**Table 3:** Pharmacokinetic parameters after performance of GNA formulations for 1.5 mg/Kg dose as in rats (*n* = 6).

Pharmacokinetic Parameters	Formulations	
	GNA Solution	GNA-Loaded PMs
C_max_ (mg L^−1^)	5.77 ± 2.26	15.89 ± 6.86 *
AUC_(0-t)_ (mg L^−1^∙min^−1^)	97.64 ± 20.50	254.85 ± 67.31 *
AUC_(0-∞)_ (mg L^−1^∙min^−1^)	116.05 ± 21.59	287.53 ± 98.80 *
MRT_(0-t)_ (min)	45.49 ± 8.30	33.49 ± 12.28

## Supplementary Materials

The following are available online at https://www.mdpi.com/2073-4360/11/5/820/s1, Figure S1: ^1^H NMR spectrum of P*t*BA in CDCl_3_ (*δ*_solvent_ = 7.26 ppm), Figure S2: GPC traces of P*t*BA (a) and P*t*BA-*b*-PCL (b), Figure S3: IR spectra of PAA-*b*-PCL (a) and P*t*BA-*b*-PCL (b).
