# Supplementary material for: Amphiphilic Block Copolymer Poly (Acrylic Acid)-B-Polycaprolactone as a Novel pH-sensitive Nanocarrier for Anti-Cancer Drugs Delivery: In-vitro and In-vivo Evaluation
Source: Polymers (Basel). 2019 May 7;11(5):820. doi: 10.3390/polym11050820 (PMC6572073; doi:10.3390/polym11050820)
Supplement: Supplementary file 1 [file polymers-11-00820-s001.pdf]

Supplementary material

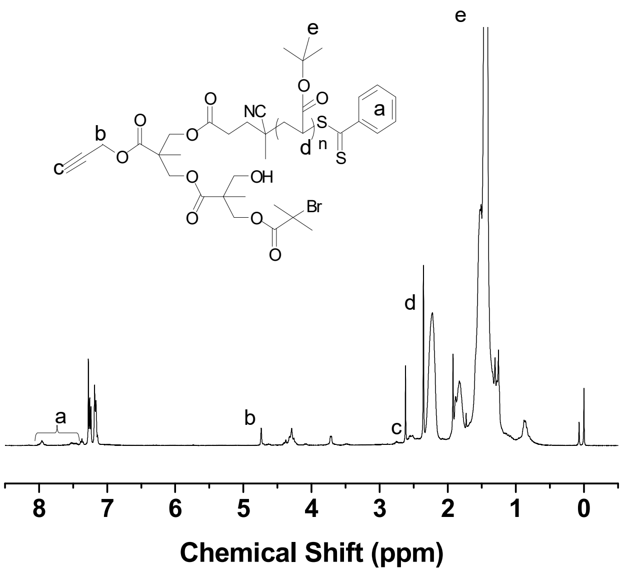

Fig. S1. ^{1}H NMR spectrum of P*t*BA in $CDCl_3$ ($\delta_{solvent}$ = 7.26 ppm).

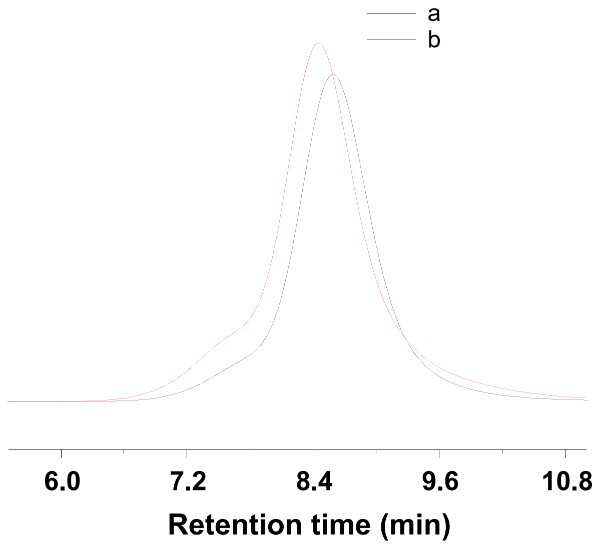

Fig. S2. GPC traces of P*t*BA (a) and P*t*BA-*b*-PCL (b).

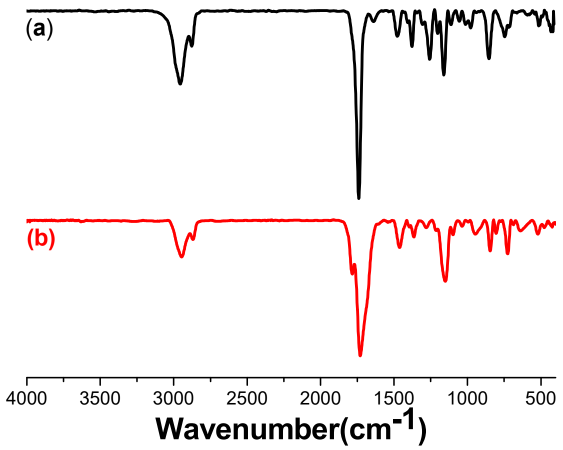

Fig.S3. IR spectra of PAA-*b*-PCL (a) and P*t*BA-*b*-PCL (b).
